# Risk factors for unsuccessful removal of central venous access ports implanted in the forearm of adult oncologic patients

**DOI:** 10.1007/s11604-021-01214-5

**Published:** 2021-11-15

**Authors:** Mitsuhiro Kinoshita, Shoichiro Takao, Junichiro Hiraoka, Katsuya Takechi, Yoko Akagawa, Kyosuke Osaki, Norio Ohnishi, Hayato Tani

**Affiliations:** 1 Department of Radiology, Tokushima Red Cross Hospital, 103, Irinokuchi Komatsushima-cho, Komatsushima, Tokushima 773-8502 Japan; 2 Department of Diagnostic Radiology, Graduate School of Health Sciences, Tokushima University, 3-18-15, Kuramoto-cho, Tokushima, Tokushima 770-8509 Japan

**Keywords:** Central venous access port, Unsuccessful removal, Indwelling period, Forearm, Adult oncologic patients

## Abstract

**Purpose:**

To evaluate the risk factors for unsuccessful removal of a central venous access port (CV port) implanted in the forearm of adult oncologic patients.

**Materials and methods:**

This study included 97 adult oncologic patients (51 males, 46 females; age range, 30–88 years; mean age, 63.7 years) in whom removal of a CV port implanted in the forearm was attempted at our hospital between January 2015 and May 2021. Gender, age at removal, body mass index, and diagnosis were examined as patient characteristics; and indwelling period, indwelling side, and indication for removal were examined as factors associated with removal of a CV port. These variables were compared between successful and unsuccessful cases using univariate analysis. Then, multivariate analysis was performed to identify independent risk factors for unsuccessful removal of a CV port using variables with a significant difference in the univariate analysis. A receiver-operating characteristics (ROC) curve was drawn for significant risk factors in the multivariate analysis and the Youden index was used to determine the optimum cut-off value for predicting unsuccessful removal of a CV port.

**Results:**

Removal of CV ports was successful in 79 cases (81.4%), but unsuccessful in 18 cases (18.6%) due to fixation of the catheter to the vessel wall. Multivariate logistic regression analysis showed that the indwelling period (odds ratio 1.048; 95% confidence interval 1.026–1.070; *P* < 0.0001) was a significant independent risk factor for unsuccessful removal of a CV port. ROC analysis showed that the cut-off value for successful removal was 41 months, and 54% of cases with an indwelling period > 60 months had unsuccessful removal.

**Conclusion:**

The indwelling period is an independent risk factor for unsuccessful removal of a CV port implanted in the forearm of adult oncologic patients, with a cut-off of 41 months.

## Introduction

Implantation of a central venous access port (CV port) was first reported in 1982 [1, 2] and is increasingly used. The indwelling period for a CV port tends to be long since the port is mainly used to administer chemotherapy for oncologic patients [3]. For these patients, removal of the port is recommended after resolution of disease or port complications such as system dysfunction or infection [3]. This removal may sometimes be problematic, with some reports describing difficulties due to fixation of the catheter to the vessel wall [4, 5]. In pediatric patients, this problem tends to occur when the indwelling period is > 20 months or the patient has a hematological disease [4]. However, to our knowledge, there have been few reports of difficulty with removal of a CV port in an adult patient [5, 6]. The purpose of this study was to evaluate the risk factors that lead to unsuccessful removal of CV ports implanted in the forearm of adult oncologic patients.

## Materials and methods

### Patients

A total of 106 consecutive patients with a solid or hematologic tumor underwent removal of a CV port at our hospital between January 2015 and May 2021. Four patients were excluded because they originally had CV port implantation at other hospitals and relevant information, including the exact date of implantation, was unknown. Five patients were excluded because they had the CV port implanted in the upper arm (*n* = 2) and chest wall (*n* = 3). Thus, 97 patients (51 males, 46 females; age, 30–88 years; mean age, 63.7 years) were included in the present study. The patient characteristics are shown in Table 1. All patients were informed about the benefits and potential risks of the CV port removal procedure, and all provided written informed consent. The institutional review board approved this retrospective study and no patient consent was required.

**Table 1:** Characteristics of patients and central venous access parts

Item	Value	Item	Value
Gender		Indwelling side	
Male	51	Left	88
Female	46	Right	9
Age at removal of CV port (years)	63.7 ± 12.7	Venous placement site	
Body mass index (kg/m^2^)	23.6 ± 5.1	Basilic	74
Indwelling period (months)	38.5 ± 36.3	Brachial	18
Diagnosis		Cephalic	5
Hematologic malignancy	72	Type of CV port system	
Lymphoma	68	DewX Eterna	24
Leukemia	2	DewX	40
Myeloma	1	BARD X-Port	8
Waldenstrom Macroglobulinemia	1	Mini Titanium Vital-Port	22
Solid malignancy	25	P-U Celsiteport	3
Pharyngeal cancer	9	Indication for removal	
Extra-auditory carcinoma	1	Complete remission of malignancy	59
Ovarian cancer	6	Complications related to CV port systems	38
Endometrial cancer	2	Possibility of infection from CV port	19
Squamous cell carcinoma	1	Catheter occlusion	8
Pancreatic carcinoma	2	Catheter breakage	5
Colorectal carcinoma	1	Catheter malposition	2
Cholangiocarcinoma	1	Other	4
Breast cancer	1		
Urachal carcinoma	1		

*CV port* central venous access port

Values are shown as number of cases or as the mean ± standard deviation

### Procedures

All CV ports were inserted under fluoroscopy and ultrasound visualization by interventional radiologists using maximum barrier precautions at our hospital. CV ports were implanted by percutaneous cannulation of basilic, brachial and cephalic veins under subcutaneous tissue of the left and right forearm. Five CV port systems were used based on the time of implantation: DewX Eterna (port size S) with a 5-Fr open-end type catheter (Terumo, Tokyo, Japan); DewX (port size S) with a 5-Fr open-end type catheter (Terumo); Bard X-Port (Bard, Salt Lake City, UT, USA) with a UK-catheter (5-Fr open-end type) (Unitika, Tokyo, Japan); Mini Titanium Vital-Port (Cook Medical, Bloomington, IN, USA) with a UK-catheter (5-Fr open-end type) (Unitika); and P-U Celsiteport with an Anthron P-U Catheter (5-Fr open-end type) (Toray, Tokyo, Japan). The details of use of each of these systems are shown in Table 1.

All CV ports were also removed under fluoroscopy by interventional radiologists. The removal procedure was as follows. (1) A skin incision was made near the area of port implantation under local anesthesia. (2) Adhesions around the port were peeled off and the port was removed. (3) The catheter connected to the port was slowly extracted under fluoroscopic guidance. (4) If the catheter could not be removed, a skin incision was made close to where the catheter entered the vein, and the catheter was pulled on directly. If these methods were ineffective, it was judged that the catheter was fixed to the vessel wall. The following methods could then be used at the operator’s discretion. (5) A guidewire could be inserted into the catheter and used to remove the catheter along with removal of the guidewire. This could only be used when there was no occlusion of the catheter. (6) The catheter movement was checked on fluoroscopy by pulling on it and to identify the adherent location. An attempt was then made to release the adherent part by covering it with a sheath, but only when the adherent location appeared to be associated with a part of the peripheral side of the catheter. (7) Venotomy of the upper arm under local anesthesia could also be used. This was performed with the assistance of vascular surgeons. In cases in which these procedures were ineffective, the catheter was cut and only the port was removed, with the catheter left in place.

### Assessments

To evaluate risk factors for unsuccessful removal of CV ports, patients with successful and unsuccessful removal were compared retrospectively. Continuous variables are expressed as mean ± standard deviation and were compared by Mann–Whitney *U* test. Categorical variables are expressed as a number and were compared by *χ*^2^ test. Gender, age at removal, body mass index (BMI), and diagnosis (hematologic or solid malignancy) were examined as patient characteristics; and indwelling period, indwelling side (left or right), and indication for removal (complete remission of malignancy or complications related to the CV port) were examined as factors associated with removal of the CV port.

Variables with a significant difference between successful and unsuccessful cases in univariate analysis were included in multivariate logistic regression analysis to identify independent risk factors for unsuccessful removal of a CV port. A receiver-operating characteristics (ROC) curve was drawn for significant risk factors in the multivariate analysis and the Youden index (sensitivity + specificity – 1) was used to determine the optimum cut-off value for predicting unsuccessful removal of a CV port. A value of *P* < 0.05 was considered to indicate statistical significance. All statistical analyses were performed using JMP ver. 13.2.1 (SAS Institute Inc., Cary, NC, USA).

## Results

Removal of CV ports was successful in 79 cases (81.4%), but was unsuccessful in 18 cases (18.6%) due to fixation of the catheter to the vessel wall.

Indwelling side (*P* = 0.0444) and indwelling period (*P* < 0.0001) were identified as significant risk factors for unsuccessful removal of a CV port in univariate analyses (Table 2).

**Table 2:** Characteristics of patients and examination parameter results

Characteristic	Total (*n* = 97)	Univariate analysis			Multivariate analysis
		Successful removal (*n* = 79)	Unsuccessful removal (*n *= 18)	*P* value	*P* value
Gender				0.1975	
Male	51 (52.6)	44 (55.7)	7 (38.9)		
Female	46 (47.4)	35 (44.3)	11 (61.1)		
Age at removal of CV port (years)	63.7 ± 12.7	64.3 ± 12.3	61.1 ± 14.3	0.3460	
Body mass index (kg/m^2^)	23.6 ± 5.1	23.9 ± 5.4	22.3 ± 3.2	0.1696	
Indwelling period (months)	38.5 ± 36.3	28.9 ± 31.8	80.6 ± 22.6	< .0001*	< .0001*
Diagnosis				0.7027	
Hematologic malignancy	72 (74.2)	58 (73.4)	14 (77.8)		
Solid malignancy	25 (25.8)	21 (26.6)	4 (22.2)		
Indwelling side				0.0444*	0.9989
Left	82 (84.5)	64 (81.0)	18 (100.0)		
Right	15 (15.5)	15 (19.0)	0 (0.0)		
Indication for removal				0.5737	
Complete remission of malignancy	59 (60.8)	47 (59.5)	12 (66.7)		
Complications related to CV port	38 (39.2)	32 (40.5)	6 (33.3)		

*CV port* central venous access port

Values are shown as the number (% valid total) of cases or as the mean ± standard deviation

*A significant difference (*P* < 0.05)

**Fig. 1:**
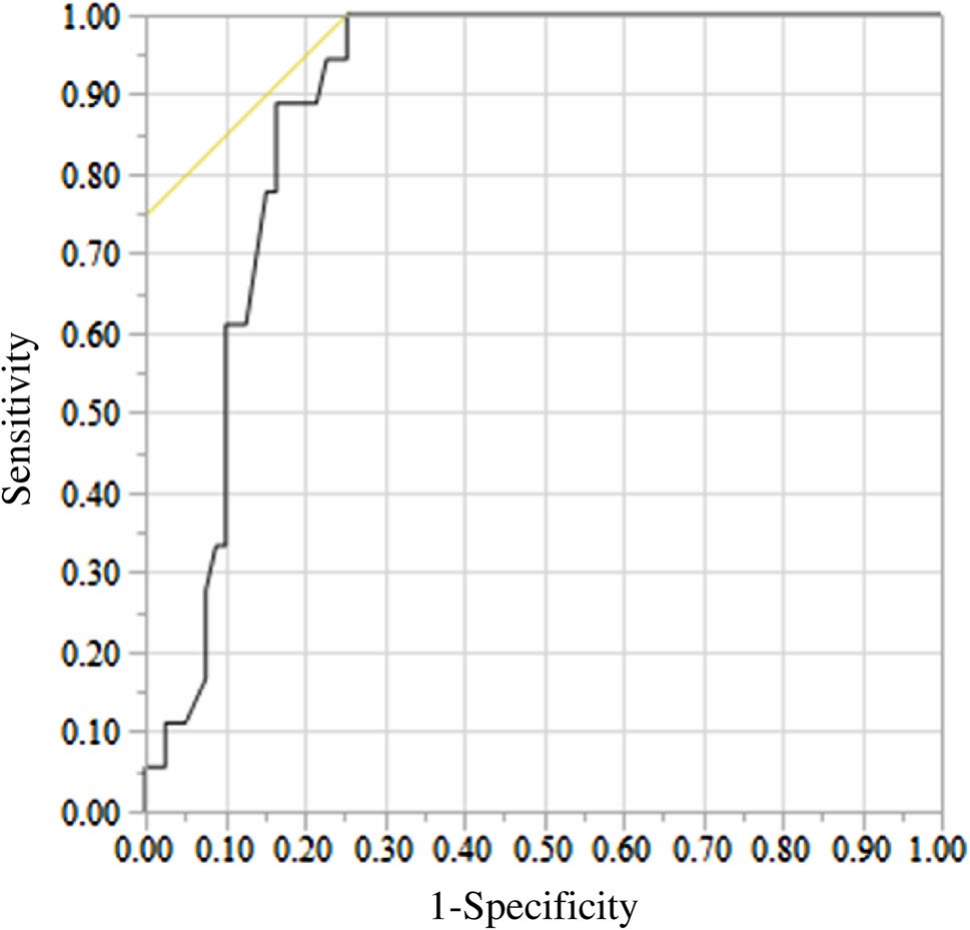
Receiver-operating characteristic curve used to determine the optimum cut-off value of the indwelling period yielding the highest combined sensitivity and specificity for predicting unsuccessful removal of a central venous access port. The cut-off was 41 months and the area under the curve was 0.886

**Fig. 2:**
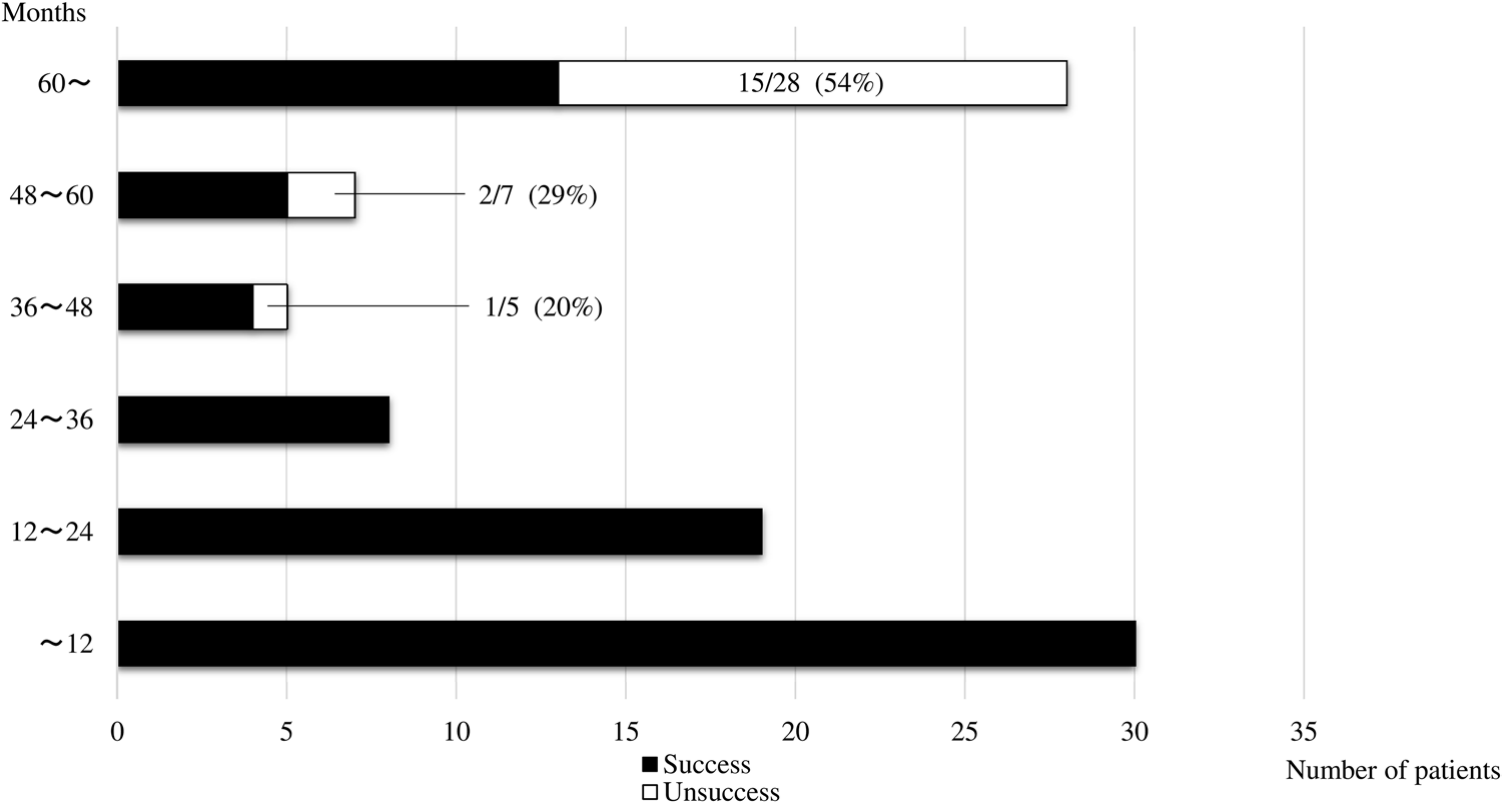
Effect of the length of the indwelling period on unsuccessful removal of a central venous access port. The incidences of unsuccessful removal were 0, 20, 29 and 54% for indwelling periods of < 36, > 36 to < 48, > 48 to < 60, and > 60 months, respectively

The indwelling period (odds ratio 1.048; 95% confidence interval 1.026–1.070; *P* < 0.0001) emerged as an independent significant risk factor for unsuccessful removal of a CV port in multivariate analysis (Table 2). ROC analysis indicated an optimum cut-off of 41 months, with an area under the curve of 0.886, sensitivity of 100%, and specificity of 75% (Fig. 1). Of cases with an indwelling period > 60 months, 54% had unsuccessful removal (Fig. 2).

A summary of patients with unsuccessful removal of CV ports is shown in Table 3. In four of the 18 cases, an attempt was made to release the adherent part by covering it with a sheath. In two of the 4 cases, venotomy of the upper arm was performed, but the catheter could not be removed. There were no symptomatic late complications in all 18 cases during each patient’s follow-up periods.

**Table 3:** Summary of patients with unsuccessful removal of central venous access parts

Patient No.	Gender	Age^*1^ (years)	BMI (kg/m^2^)	Diagnosis	Indwelling side	Venous placement site	Type of CV port	Indication for removal	Indwelling period (months)	Removal procedure^*3^				Follow-up period^*4^ (months)	Symptomatic late complication^*5^
										(1)–(4)	(5)	(6)	(7)		
1	M	66	25.4	Lymphoma	L	Basilic	DewX	CR	41	+	+			8	None
2	F	88	21.3	Lymphoma	L	Basilic	DewX	Infection^*2^	49	+	+	+		28	None
3	F	48	18.4	Leukemia	L	Basilic	Vital-Port	Other	58	+	+			60	None
4	F	49	19.2	Laryngeal Ca	L	Basilic	DewX	CR	62	+	+			7	None
5	F	42	19.9	Lymphoma	L	Brachial	X-Port	CR	70	+	+			36	None
6	F	68	25.1	Lymphoma	L	Basilic	Vital-Port	CR	70	+	+	+		36	None
7	M	77	24.1	Pharyngeal Ca	L	Basilic	Vital-Port	CR	70	+	+			33	None
8	M	45	21.5	Lymphoma	L	Basilic	Vital-Port	CR	78	+	+			23	None
9	F	71	18.3	Lymphoma	L	Basilic	X-Port	CR	80	+	+			9	None
10	M	39	23.9	Lymphoma	L	Cephalic	Vital-Port	CR	83	+	+			21	None
11	F	70	20.9	Lymphoma	L	Basilic	Vital-Port	CR	83	+	+			28	None
12	M	69	23.2	Extra-auditory Ca	L	Brachial	Vital-Port	CR	84	+	+			41	None
13	F	44	22.9	Ovarian Ca	L	Basilic	Vital-Port	CR	87	+	+			58	None
14	F	56	31.2	Myeloma	L	Basilic	Vital-Port	Occlusion	91	+				41	None
15	M	69	21.0	Lymphoma	L	Basilic	Vital-Port	CR	98	+	+			2	None
16	F	53	19.7	Lymphoma	L	Basilic	Vital-Port	Occlusion	99	+	+	+	+	54	None
17	M	67	24.6	Lymphoma	L	Basilic	Celsite port	Infection^*2^	109	+	+	+	+	10^♰^	None
18	F	78	20.0	Lymphoma	L	Basilic	Vital-Port	Breakage	138	+				3	None

*M* male, *F *female, *BMI* body mass index, *Ca* carcinoma, *L* left, *CV port* central venous access port, *CR* complete response *Vital-Port *Mini Titanium Vital-Port, *X-Port *BARD X-Port, *Celsiteport* P-U Celsiteport

^*1^Age at removal of CV port

^*2^Possibility of infection from CV port

^*3^Refer to Materials and methods in the text for details of the removal procedure in (1)–(7)

^*4^The period after attempt to remove the CV port

^*5^The complication related to the catheter left in place

^♰^Died due to aggravated underlying disease

## Discussion

There have been many reports of complications related to implantation or use of CV ports [3, 7], but few of problems during removal of these ports, due to the lower frequency of removal compared to implantation. The most commonly reported problem is the difficulty of catheter removal in the CV port system. In the present study, there were cases in which the CV port adhered strongly to tissue under the skin. The port could be removed in all cases, but the catheters could not be removed in 18.6% of the cases. In the pediatric study also referred to above [4], the rate of difficulty of removal was 16%, and in 4% of cases venotomy was required for catheter removal or the catheter was left in place. Factors that contributed to a higher risk of removal difficulty included the indwelling period and hematological diseases, and all cases with removal difficulties had an indwelling period of > 20 months [4].

In the present study, the indwelling period was the only risk factor in multivariate analysis, with a cut-off value of 41 months, and 54% of cases with an indwelling period of > 60 months had unsuccessful removal. The phenomenon of fixation to the vessel wall as a cause for catheter retention has been previously described, and the rate of unsuccessful removal of a central venous catheter (CVC) in pediatric patients is 0.2–2.0% [8–11]. In a series of 136 procedures for CVC removal, Jones et al. identified 7 cases with difficulty related to fixation of the catheter [11]. In 3 of these cases the catheter was left in place, and 2 of these cases were patients with acute lymphoblastic leukemia (ALL). Implanted CV ports in cases with hematological diseases have been found to have a significantly greater risk of difficulty of catheter removal, and ALL is the commonest diagnosis within this group [4]. It is possible that certain chemotherapeutic agents may contribute to the process causing catheter fixation. In the present study, there were only 2 cases with leukemia, and this small number is likely to account for hematological diseases not emerging as a risk factor for unsuccessful removal of a CV port.

Histological changes caused by prolonged catheterization have been investigated in use of CVCs [12, 13]. Focal damage to the vein intima adjacent to the catheter causes organizing thrombi, collagen, and surface reendothelialization, and this damage occurs after a relatively short indwelling period. Over a longer period, these effects lead to vein wall thickening, formation of bridges from the vein wall to the catheter, and the appearance of prominent fibrin sheaths surrounding the catheter. These sheaths contain collagenous tissue, fibrin, or tissue including endothelial cells. The catheter becomes firmly fixed to the vein wall and is difficult to remove after prolonged implantation. These observations constitute a progressive reaction of the walls of human veins to catheters. Thus, the reason for the higher rate of unsuccessful removal of catheters in the present study compared with the pediatric study [4] is likely to be the longer mean indwelling period (40 months) compared to that in the pediatric study (29 months). If there is a drug leakage due to catheter breakage prior to catheter removal, it is possible that inflammatory adhesions due to the leakage may make removal difficult. In the present study, there were only 2 cases with catheter breakage, and we could not fully analyze these issues due to the small number of cases.

The optimum cut-off for successful catheter removal in the present study (41 months) was also longer than that in the pediatric study (20 months) [4]. This may be because the diameter of the vein in which the catheter was placed in the pediatric study was smaller than that in the present study. The quickness of fixation to the vein wall in the pediatric study could be a result of the percentage area of contact of the catheter with the vein wall. This area is much higher in pediatric patients than in adults due to the difference in vein width.

If the catheter is left in the body, delayed complications such as infection and thrombosis may occur. In the present study, there were no symptomatic late complications during each patient’s follow-up periods, but careful follow-up is necessary.

The present study is limited by four factors: first, the retrospective design and the relatively low incidence of unsuccessful removal of CV ports, which limits the statistical power. Second, few patients with unsuccessful removal had less common variables, and this precluded analysis of the diagnosis and indication for removal as risk factors for unsuccessful removal. In particular, evaluation of more cases with leukemia and catheter breakage would be ideal. Third, effects of the type of CV port system, including the catheter material, and the chemotherapeutic regimens were not evaluated because of the wide variety used in the present study. Forth, since this study has focused on CV ports implanted in the forearm, the results may not be directly applied to CV ports implanted in other locations. Thus, studies of these effects in larger cohorts would be beneficial for further verification of the risk factors for unsuccessful CV port removal.

In conclusion, the indwelling period was an independent risk factor for unsuccessful removal of CV ports implanted in the forearm of adult oncologic patients, with a cut-off of 41 months. Of patients with an indwelling period > 60 months, 54% had unsuccessful removal. However, further research is needed to confirm these results because of the small number of cases of unsuccessful removal and the lack of variation of CV port characteristics in the study.
